# 17β‐estradiol ameliorates age‐associated loss of fibroblast function by attenuating IFN‐γ/STAT1‐dependent miR‐7 upregulation

**DOI:** 10.1111/acel.12462

**Published:** 2016-03-02

**Authors:** Adam C. Midgley, Glyn Morris, Aled O. Phillips, Robert Steadman

**Affiliations:** ^1^ Institute of Nephrology, Wales Kidney Research UnitDivision of Infection and ImmunityCardiff UniversityCollege of Biomedical and Life SciencesUniversity Hospital of WalesHeath ParkWalesCF14 4XNUK

**Keywords:** 17β‐estradiol, fibroblast, interferon‐γ, microRNA‐7, myofibroblast, wound healing

## Abstract

Age‐related defects in fibroblast differentiation and functionality were previously shown to be associated with impaired hyaluronan (HA) synthase 2 (HAS2) and epidermal growth factor receptor (EGFR) function, as a result of upregulated microRNA‐7 (miR‐7) expression. In aging fibroblasts, inhibiting miR‐7 prevented the dysregulation of the HA‐mediated CD44/EGFR signaling pathway. Here, we investigated transcriptional upregulation of miR‐7 and implicated the age‐associated over‐activation of JAK/STAT1 as a primary candidate. STAT1 binding sites were identified on the putative miR‐7 promoter and stimulation of fibroblasts with the inflammatory cytokine, interferon‐γ (IFN‐γ), significantly increased miR‐7 transcriptional activity and resulted in upregulated miR‐7 and loss of EGFR. Additionally, we demonstrated a role for the anti‐inflammatory steroid, 17β‐estradiol (E2), in the attenuation of miR‐7 expression. E2 stimulation promoted estrogen receptor (ER) interactions with the miR‐7 putative promoter and suppressed miR‐7 expression. E2 also attenuated STAT1 expression and activity. Furthermore, treatments with E2 restored fibroblast functionality, including proliferation, migration and differentiation, key events in effective wound healing. In light of our findings, we propose that the regulation of miR‐7 by pro‐ and anti‐inflammatory mediators plays a wider role than previously thought. The modulation of fibroblast functions and ultimately wound healing by miR‐7 activators or inhibitors could provide realistic targets for the restoration of chronic wound healing capabilities in the elderly.

## Introduction

Fibroblasts are central to remodeling and restoration of tissue integrity during wound healing. Differentiation [triggered by transforming growth factor (TGF)‐β1], into α‐smooth muscle actin (αSMA)‐positive, contractile myofibroblasts (Gabbiani, 2003), allows for wound closure and the formation of collagen‐rich scars. Fibroblasts isolated from aging skin, however, show premature senescence, impaired migration, proliferation and matrix generation (Campisi, 1998; Stephens *et al*., 2003). Our recent studies showed aging fibroblasts were resistant to TGF‐β1 differentiation (Midgley *et al*., 2013) and this may be an important defect of wound healing in the elderly. In young fibroblasts, TGF‐β1 activates two distinct, co‐operating pathways. The first is the TGF‐βR/Smad2‐mediated signaling pathway. The second is mediated by hyaluronic acid (HA) and its receptor CD44. HA‐mediated CD44 translocation within the plasma membrane allows for co‐localization with epidermal growth factor receptor (EGFR) in lipid rafts. This directs intracellular signaling through extracellular signal‐regulated kinases (ERK1/2). In aged cells, the EGFR pathway is dysregulated due to reduced expression and impaired CD44/EGFR co‐localization, while the Smad2 pathway remains intact. We have shown more recently that in aged fibroblasts, reduced EGFR expression resulted from increased expression of microRNA (miR)‐7 (Midgley *et al*., 2014). Reducing miR‐7 expression restored EGFR and the TGF‐β1 differentiation response. In contrast, overexpression of miR‐7 in young fibroblasts decreased EGFR expression and the TGF‐β1 response. This novel mechanism of a miR‐7‐dependent and age‐associated functional effect on differentiation highlights miR‐7 as a potential target for restoring fibroblast functionality and, by implication, chronic wound healing in the elderly.

Upregulation of miR‐7 was reported to involve c‐Myc binding the miR‐7 promoter and enhancing transcriptional activity. This was dependent on the activation of EGFR‐regulated ERK/c‐Myc (Chou *et al*., 2010). In addition, miR‐7 has been reported to target Akt and blockade of the EGFR‐mediated PI3K/Akt pathway resulted in attenuated miR‐7 expression (Fang *et al*., 2012). These studies highlight two self‐regulatory loops for miR‐7 expression. In aging fibroblasts, the expression of EGFR is lost, suggesting pathways involving EGFR‐dependent c‐Myc or Akt would not be active. Therefore, upregulated expression of miR‐7 in aged fibroblasts is likely to be through an alternative, constitutively active pathway.

Interestingly, the inflammatory mediators, interferon‐γ (IFN‐γ) and interleukin‐6 (IL6), which primarily signal through the STAT1 and STAT3 pathways, respectively, were found to have increased expression in aged mice (Busse *et al*., 2007). Furthermore, studies have correlated aging with increased expression of inflammatory mediators, specifically following menopause or andropause (Ershler & Keller, 2000; Tchkonia *et al*., 2010). The findings that inflammatory regulators are present at elevated levels in aged individuals could provide a pivotal role for the immune system in the progression or cessation of successful wound healing. Tumor necrosis factor‐α (TNF‐α) is a pro‐inflammatory cytokine involved in leukocyte recruitment, M1/M2 macrophage shifts and delaying wound healing responses. Blockade of TNF‐α increased matrix synthesis and accelerated healing in murine models of age‐related impaired healing and excessive inflammation (Ashcroft *et al*., 2012). IL6 can display pro‐ and anti‐inflammatory actions: IL6‐KO mice exhibit impaired granulation tissue formation and delayed cutaneous wound healing; IL6‐KO dermal fibroblasts had decreased production of matrix enzymes, suggesting an IL6 role in modulating fibroblast function (Luckett & Gallucci, 2007). One later study demonstrated that IL6Rα‐ERK signaling improved wound healing (McFarland‐Mancini *et al*., 2010). In contrast to IL6, IFN‐γ decreased formation of new granulation tissue in rats and dysregulated collagen production and *in vitro* experiments demonstrated that IFN‐γ decreased collagen synthesis in rat fibroblasts (Laato *et al*., 2001). IFN‐γ‐KO mice displayed accelerated collagen deposition, granulation tissue formation and wound healing, subsequent to increased TGF‐β1 activity and decreased IFN‐γ/STAT1 activation (Ishida *et al*., 2004). These studies highlight the differential roles of the immune response in wound healing, whether these cytokines modulate fibroblast function through EGFR changes, has yet to be investigated.

Previous research has shown that treatment with the anti‐inflammatory and estrogen‐derived 17β‐estradiol (E2) attenuated the age‐related increase in inflammatory mediators (Girasole *et al*., 1992; Ray *et al*., 1997). Estrogen‐treated splenocytes had reduced levels of IFNγ production and modulated STAT1 binding activity, in inflammatory models (Dai *et al*., 2009). A link between E2 and aging has already been established: Differences in gene expression between young and aged wound biopsies were found to be largely estrogen‐regulated (Hardman & Ashcroft, 2008). Furthermore, E2 accelerated wound healing in ovariectomized mice (Hardman *et al*., 2008). E2 has also been shown to have a role in the regulation of EGFR and accelerated wound healing (Stabile *et al*., 2005; Tsonis *et al*., 2013). E2 enters cells freely and interacts with cytoplasmic target cell receptors, estrogen receptor‐α (ERα) and estrogen receptor‐β (ERβ). The ER‐complex can enter the nucleus of the target cell (Levin, 2005) and regulate gene transcription through the modulation of co‐transcription factors or by direct binding of DNA. Changes in ER and EGFR expression followed similar profiles under E2 treatments and in inhibition studies, suggesting co‐regulation (Koibuchi *et al*., 2000). Furthermore, the combined inhibition of ER and EGFR caused antiproliferative effects in cancer cells (Stabile *et al*., 2005). However, in MCF‐7 breast cancer cells, E2 treatments were detrimental to EGFR expression in an ERα‐ and miR‐7‐dependent manner (Masuda *et al*., 2012).

In this study, we investigated miR‐7 transcription through the analysis of its promoter region and identification of potential transcription factors. We report a role for IFNγ and STAT1 activation in the upregulation of miR‐7 transcriptional activity and expression in fibroblasts. We also investigated the effect of E2 on gene expression and function in young and aged fibroblasts, involving changes to STAT1 activation and modulation of its binding capacity. Finally, we examined whether combinational treatments of TGF‐β1 with E2 could restore the differentiation potential in aged fibroblasts.

## Results

**Figure 1:**
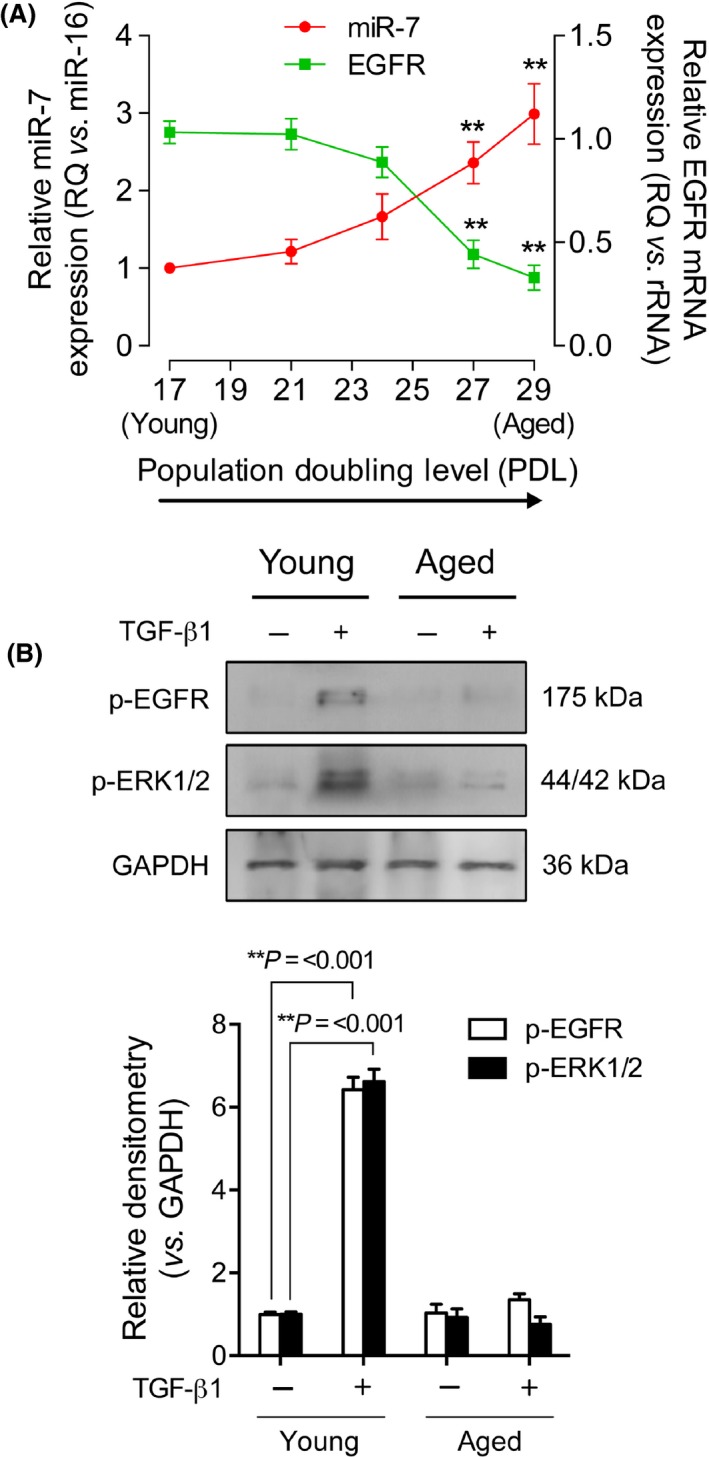
Increasing fibroblast population doubling level (PDL) correlates with elevated miR‐7 expression and decreased epidermal growth factor receptor (EGFR) expression. (A) qPCR for miR‐7 (red circles) and EGFR (green squares) mRNA expression (**P = <0.01 when compared to PDL 17 (young) cultures). (B) Western blots to assess p‐EGFR (open bars) and p‐ERK1/2 (black bars). GAPDH was used as a loading control and data are normalized to young, untreated fibroblasts. Images are representative of three blots, and all data are displayed as means ± SEM of three independent experiments. **P = <0.01.

Previously, we have shown *in vitro* that fibroblasts with high population doubling levels (PDL) had low EGFR expression due to an age‐associated increase in miR‐7 expression (Midgley *et al*., 2014). In this study, we have investigated the relationship between EGFR and miR‐7 further. At a threshold of approximately PDL >24, EGFR expression declined and miR‐7 expression began to increase. Beyond PDL 27‐29 (aged), mRNA expression levels of EGFR and miR‐7 were significantly different to PDL 17 (young) fibroblasts (Fig. 1A). Analysis of phosphorylation (p‐) states of EGFR and ERK1/2 by TGF‐β1 demonstrated a loss of signaling capacity in aged fibroblasts (Fig. 1B). The downregulation of EGFR by miR‐7 impacted on the signaling kinase, ERK1/2, which is involved in an array of functional pathways essential in wound healing: proliferation, migration and differentiation.

**Figure 2:**
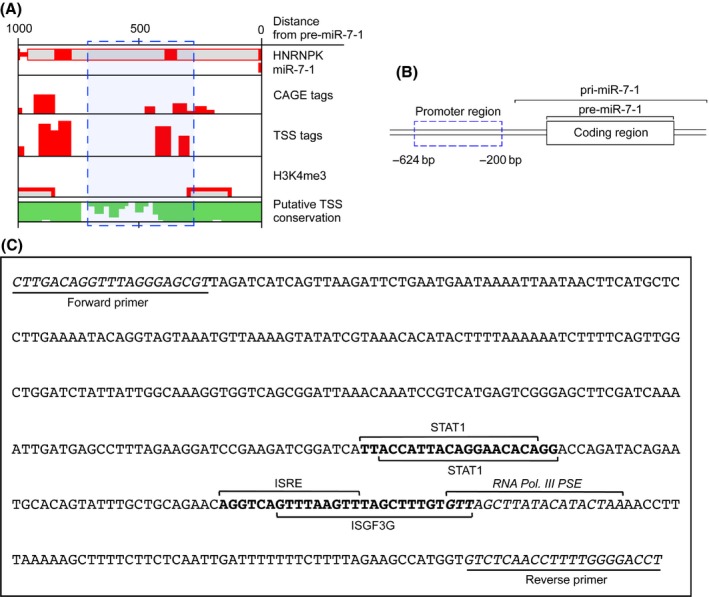
Analysis of the miR‐7 promoter reveals STAT binding sites. *In silico* analysis was used to determine (A) the putative transcription start site (TSS) for miR‐7‐1 (blue box indicates region of predicted TSS, as indicated by flanking CAGE tags and H3K4me3 regions). Image was generated and analyzed using miRStart (Institute of Bioinformatics and Systems, National Chiao Tung University, Hsinchu, Taiwan). (B) Diagram of miR‐7‐1 coding region and promoter region used for analysis (dashed blue box). (C) The promoter region for miR‐7‐1 was analyzed for recognized transcription factor‐binding sites using MatInspector (Genomatix Software). Transcription factors of interest are labeled, and underlined sections indicate the primers used for promoter amplification in subsequent experiments.

Binding of miR‐7 to highly conserved EGFR‐3′ UTR (Midgley *et al*., 2014) results in the degradation of EGFR Mrna, and therefore, translation to protein no longer transpires. Previous reports indicated that the miR‐7 promoter extends to −600 bp upstream of the pre‐miR‐7 coding region (Chou *et al*., 2010). *In silico* analysis of the putative transcription start site (TSS) for pri‐miR‐7 determined its location to be approximately −250 bp upstream of the miR‐7 coding region, as indicated by the density of highly conserved cap analysis gene expression (CAGE) tags and TSS tags flanked by histone‐3 lysine‐4 trimethylation (H3K4me3) sites (Fig. 2A). Therefore, a sequence −624 bp to −200 bp upstream of the coding region (Fig. 2B) was analyzed *in silico* for putative transcription factor‐binding sites (Fig. 2C). Multiple STAT binding sites were found, including sites for STAT1, IFN‐stimulated regulatory element (ISRE) and IFN‐stimulated transcription factor 3‐γ (ISGF3G). These were situated upstream of a likely TSS location as indicated by RNA Pol. III PSE, a strong determinant for the recruitment of RNA polymerase III (Schramm & Hernandez, 2002). These potential transcription factor‐binding sites strongly suggested that STAT activation could contribute to the upregulated expression of miR‐7.

**Figure 3:**
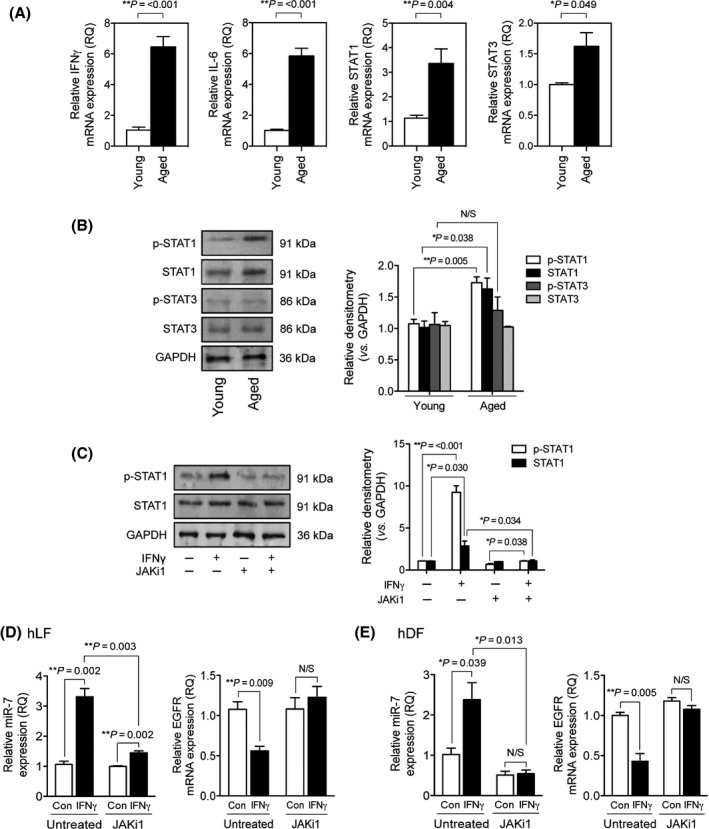
Inflammatory regulators are increased in aging fibroblasts and miR‐7 induction is downstream of JAK/STAT1. (A) qPCR analysis of interferon‐γ (IFNγ), IL‐6, STAT1 and STAT3 mRNA expression. (B) Western blots to assess total and p‐STAT1/STAT3. GAPDH was used to ensure equal loading. (C–E.) Cells were pretreated with 10 μm JAK inhibitor 1 (JAKi1) for 1 h prior to 72 h of incubation in serum‐free (SF) media, alone or containing 10 ng mL^−1^ IFNγ. (C) Western blot analysis of p‐STAT1 (open bars) and total STAT1 (black bars). GAPDH ensured equal gel loading. Expression of miR‐7 and epidermal growth factor receptor (EGFR) mRNA in (D) hLF and (E) hDF was analyzed by qPCR. Blots are representative of three individual gels, and all results are displayed as mean ± SEM of three separate experiments. **P = <0.01 and *P = <0.05.

We investigated whether the mRNA expression of IFNγ and IL6 and downstream signaling proteins altered with cellular age, as was previously suggested (Ershler & Keller, 2000; Busse *et al*., 2007). IFNγ, IL6, STAT1 and STAT3 mRNA were all significantly expressed at higher levels in aged fibroblasts (Fig. 3A). Western blots were used to assess activation levels of STAT1 and STAT3 (Fig. 3B). STAT1 exhibited strong phosphorylation in aged fibroblasts; however, there was no significant difference observed in p‐STAT3 levels between aged and young fibroblasts. Interestingly, there was a significant increase in the total STAT1 protein present in aged cells. These observations were likely due to increased IFNγ production potentiating STAT1 and p‐STAT1 levels. Despite the increase in IL6 mRNA expression, activation of STAT3 did not increase, suggesting the miR‐7 changes may be dependent on the phosphorylation of STAT1 rather than STAT3.

To investigate the role of the JAK/STAT1 pathway in miR‐7 transcription, JAK activation was inhibited using JAK inhibitor 1 (JAKi1) and Western blot was used to determine p‐STAT1 as an outcome (Fig. 3C). IFNγ strongly phosphorylated STAT1 and increased total STAT1 protein expression by 4‐fold. Treatment with JAKi1 prevented these increases. The expression of miR‐7 and EGFR in human lung fibroblasts (hLF) (Fig. 3D) and human dermal fibroblasts (hDF) (Fig. 3E) was then examined by qPCR. In both types of fibroblast, IFNγ upregulated miR‐7 expression significantly. EGFR mRNA expression was downregulated by 50%. JAKi1 significantly inhibited the IFNγ induction of miR‐7 and completely prevented the reduction of EGFR expression.

**Figure 4:**
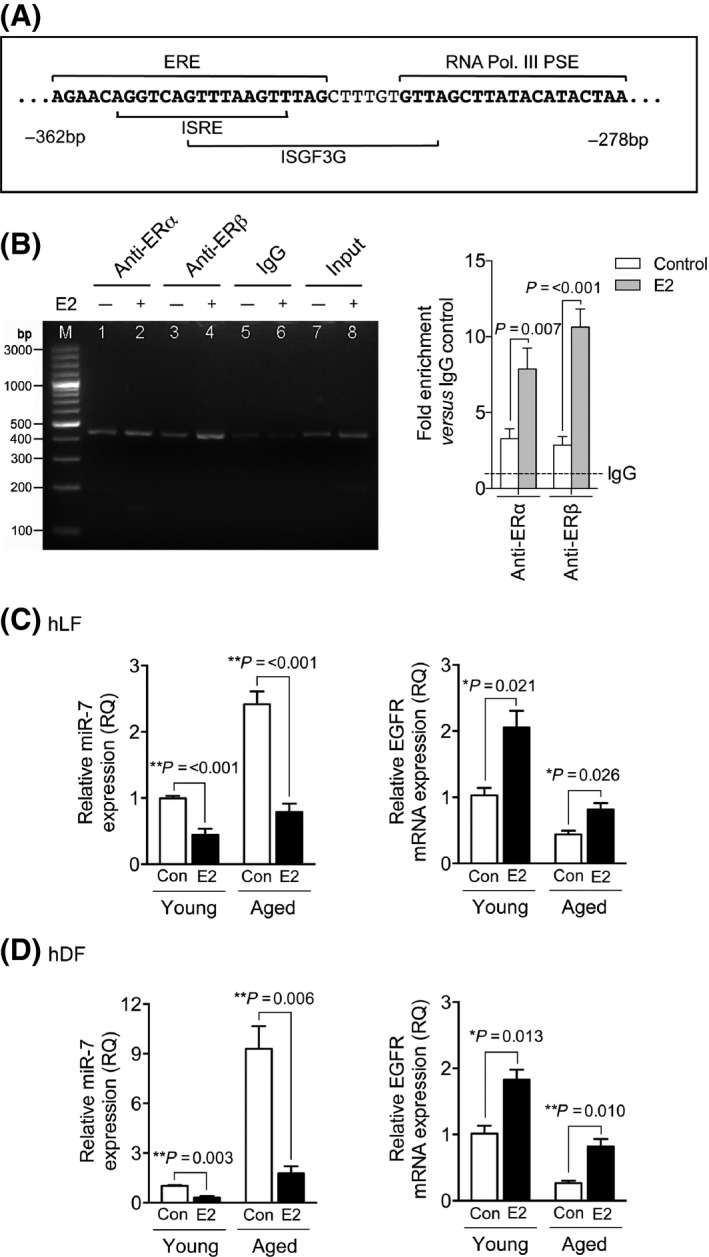
Estrogen receptors bind to the miR‐7 promoter and suppress miR‐7 expression. (A) Immediate nucleotide sequence upstream of the RNA polymerase III proximal sequence element (RNA Pol. III PSE); ER‐activated estrogen response element (ERE) is indicated in bold. (B) Young fibroblasts were incubated with serum‐free (SF) medium alone (open bars) or 10^−7^ m E2 (gray bars) for 24 h. Following treatments, ERα/ERβ association with miR‐7 promoter was assessed by ChIP (IgG = negative control ChIP; Input = starting sonicated DNA). Cells were incubated in SF medium alone (white bars) or SF medium containing 10^−7^ m E2 (black bars) for 72 h and expression of miR‐7 and epidermal growth factor receptor (EGFR) mRNA in (C) hLF and (D) hDF was analyzed by qPCR. Results are shown as mean ± SEM of three individual experiments. *P = <0.05, **P = <0.01.

*In silico* analysis was used to identify a potential suppressor of miR‐7 transcription. The presence of an ER‐activated estrogen response element (ERE) was identified within 10 bp of the RNA Pol. III PSE and overlapped the ISRE and ISGF3G binding sites (Fig. 4A). To determine whether this ERE site was responsible for the regulation of miR‐7 transcription, E2 – a prominent activator of ER – was used to stimulate fibroblasts. Chromatin immunoprecipitation (ChIP) was used to investigate ER association with the miR‐7 promoter (Fig. 4B). Both ERα and ERβ had an increased association with the miR‐7 promoter, under E2 stimulation. The effects of ER binding to the miR‐7 promoter region were assessed by qPCR for miR‐7 and EGFR expression in hLF (Fig. 4C) and hDF (Fig. 4D). Treatment with E2 resulted in an attenuation of miR‐7 and increased EGFR mRNA expression in both young and aged fibroblasts. These results suggested that ER binding to ERE within the miR‐7 promoter had a repressive role in miR‐7 transcriptional regulation, affecting the intracellular miR‐7 levels, leading to reciprocal increases in EGFR mRNA expression.

**Figure 5:**
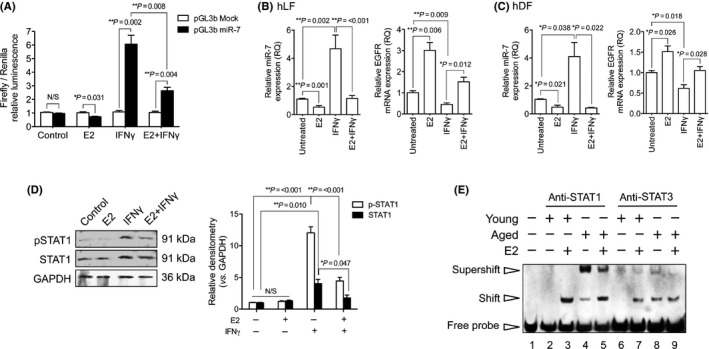
Transcriptional activity of miR‐7 is strongly induced by interferon‐γ (IFNγ)/STAT1 and attenuated by E2. (A) Fibroblasts were transfected with empty pGL3b vector (white bars) or pGL3b‐miR‐7 (black bars). Cells were incubated in serum‐free (SF) media alone, or SF media containing either 10^−7^ m E2, 10 ng mL^−1^ IFNγ or 10^−7^ m E2 with 10 ng mL^−1^ IFNγ for 24 h. Firefly luciferase was measured using a luminescence plate reader. Renilla luciferase was co‐transfected for efficiency and normalization. qPCR was used to assess miR‐7 and epidermal growth factor receptor (EGFR) mRNA in (B) hLF and (C) hDF. (D) Western blots for total and p‐STAT1. GAPDH ensured equal protein loading. E. Electromobility shift/supershift assay (EMSA) using biotinylated miR‐7 promoter DNA investigated binding potential of proteins. Supershift assay used STAT1 and STAT3 antibodies to identify specific protein–promoter interactions. Images shown are representative of three individual gels. All results are displayed as mean ± SEM of three individual experiments. Immunoblots are representative of three experiments. *P = <0.05, **P = <0.01.

The anti‐inflammatory effects of E2, including actions on IFN‐γ, have been extensively investigated (Ray *et al*., 1997; Dai *et al*., 2009; Akabori *et al*., 2010). To assess differential effects of E2 and IFNγ on miR‐7 promoter activation and the STAT pathway, fibroblasts were transfected with empty pGL3b or miR‐7‐pGL3b. There was a significant, 7‐fold induction of luciferase activity stimulated by IFNγ, compared to vector controls (Fig. 5A). E2 treatment generated a significant 40% reduction in luciferase activity. In cells that were treated with E2 + IFNγ, the IFNγ luciferase response was inhibited by 60%. E2 also significantly antagonized IFNγ induction of miR‐7 and EGFR mRNA expression in hLF (Fig. 5B) and hDF (Fig. 5C). EGFR mRNA following these treatments was inversely correlated to miR‐7 expression, as expected: IFNγ significantly reduced EGFR expression by approximately 50%, and the combination of E2 + IFN‐γ resulted in an attenuation of the IFNγ downregulation. Western blot analysis (Fig. 5D) indicated that E2 + IFNγ attenuated total STAT1 and p‐STAT1 protein, suggesting an additional role for E2 in inhibiting STAT1 expression. Further analysis using electromobility shift/supershift assay (EMSA) determined whether E2 treatment affected STAT1 association with the miR‐7 promoter, through competitive ER binding (Fig. 5E). DNA shifts indicated that there was no constitutive binding of nuclear protein to the probe (lanes 2 and 6). In contrast, protein–probe interactions increased (shift) in aged fibroblasts (lanes 4 and 8). Supershifts were present when either anti‐STAT1 (strong supershift; lane 4) or anti‐STAT3 (weak supershift; lane 8) was added to the reactions. E2 treatment resulted in an observable shift (lanes 3, 5, 7 and 9); however, there was a noticeable reduction in the STAT1 supershift seen in aged fibroblasts (lane 5). These supershift assays demonstrated that STAT1 had strong association with the putative promoter region of miR‐7, whereas STAT3 was weakly associated under aged and E2 conditions. These results suggested E2‐induced ER may directly reduce the binding capacity of STAT1 to the same promoter region.

**Figure 6:**
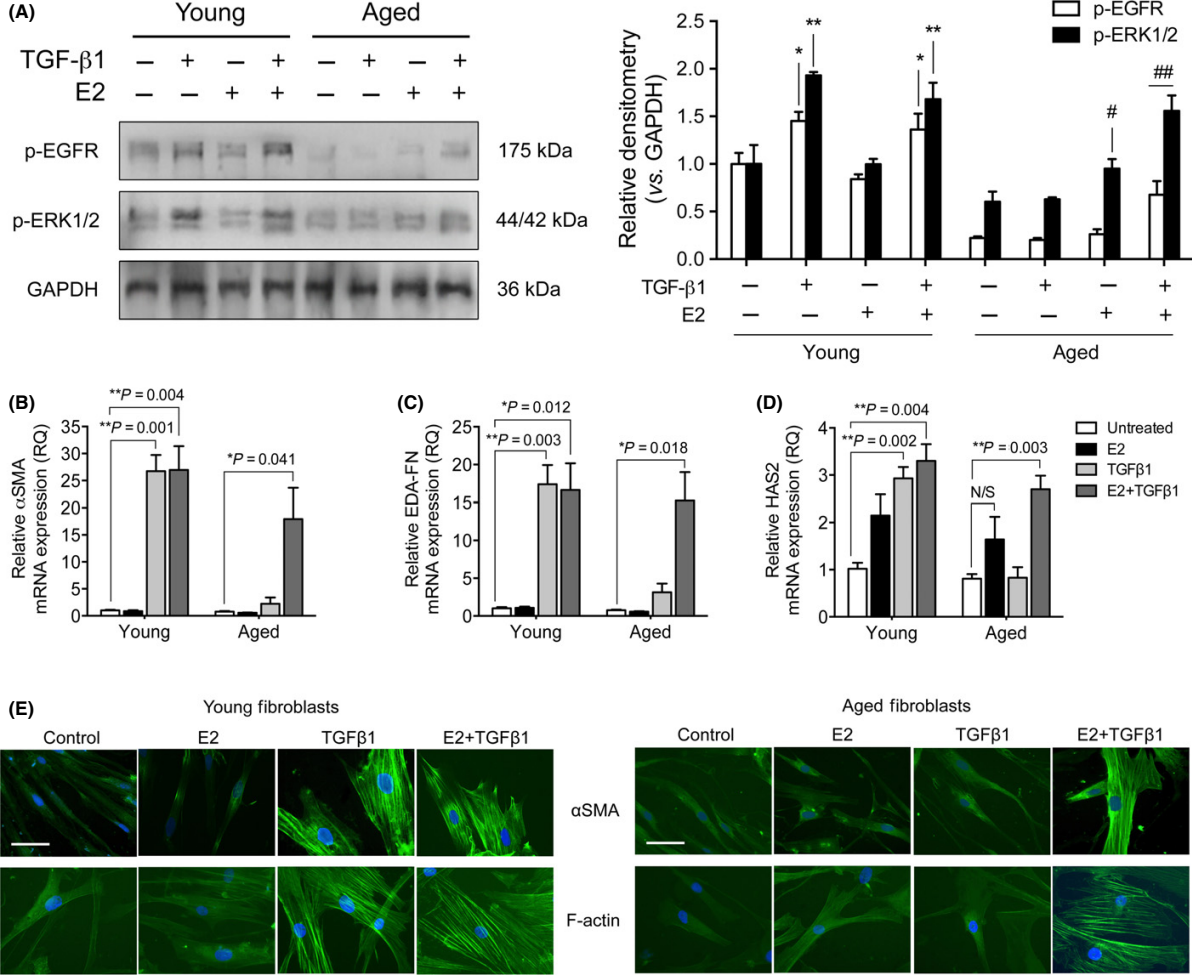
E2 rescues the transforming growth factor (TGF)‐β1 differentiation response in aging fibroblasts. (A) Western blot was used to assess p‐EGFR (open bars) and p‐ERK1/2 (black bars) in fibroblasts treated with TGF‐β1 and/or E2 for 24 h. GAPDH was used as a loading control, and data were normalized to young, untreated fibroblasts. Images are representative of three blots (*P = <0.05/**P = <0.01 – significance against young, untreated; ^#^ *P* = <0.05/^#^^#^ *P* = <0.01 – significance against aged, untreated). Cells were incubated with SF medium (white bars), 10^−7^ m E2 (black bars), 10 ng mL^−1^ TGF‐β1 (light gray bars) or 10^−7^ m E2 and 10 ng mL^−1^ TGF‐β1 (dark gray bars) for 72 h. qPCR was used to assess mRNA expression of (B) αSMA, (C) EDA‐FN, and (D) HAS2. Results are shown as mean ± SEM of three individual experiments. *P = <0.05, **P = <0.01. (E) Cells were stained for α‐smooth muscle actin (αSMA) (top row, each panel) or F‐actin (bottom row, each panel) and visualized using fluorescence microscopy. Images are representative of five individual experiments. White scale bars represent 50 μm.

In light of the findings that E2 treatment was able to suppress miR‐7 expression, we sought to investigate the potential of E2 stimulation to influence aged fibroblast capacity to respond to TGF‐β1. Firstly, p‐EGFR and p‐ERK1/2 levels in young and aged fibroblasts were analyzed by Western blot (Fig. 6A). In young fibroblasts, TGF‐β1 treatment resulted in a significant upregulation of p‐EGFR and p‐ERK1/2. In aged fibroblasts, TGF‐β1 was able to significantly increase p‐EGFR and p‐ERK1/2 only when in the presence of E2. These results indicated that E2 treatment restored p‐EGFR levels to a sufficient threshold to allow for the phosphorylation of ERK1/2. Restoration of EGFR‐ERK1/2 suggested E2‐treated aged fibroblasts would be able to respond to TGF‐β1‐driven differentiation. Therefore, qPCR analysis measured the mRNA expression of differentiation markers: αSMA (Fig. 6B), EDA‐FN (Fig. 6C), and HAS2 (Fig. 6D). In young fibroblasts, the expression of the three markers increased with TGF‐β1 stimulation. In aged fibroblasts, significantly increased mRNA expression levels were observed for all three markers under combined treatment with TGF‐β1 and E2. To confirm myofibroblast differentiation, immunocytochemistry was used to stain for αSMA stress fibers and F‐actin rearrangement (Fig. 6E). In young fibroblasts, TGF‐β1 treatment resulted in the formation of αSMA stress fibers and F‐actin bundles throughout the cell cytoplasm. When E2 was used with TGF‐β1 to stimulate aged fibroblasts, αSMA stress fibers and F‐actin were detectable, unlike in the other treatment conditions. These results suggested that E2‐treated aged fibroblasts could undergo TGF‐β1‐driven differentiation to phenotypes indicative of myofibroblasts.

To confirm that the changes in marker expression were correlated with restoration of EGFR‐associated function, we sought to assess proliferation (Table 1; Fig. S1A–B, Supporting information), migration (Table 1; Fig. S1C–D, Supporting information), and contraction (Table 2; Fig. S2A–B, Supporting information). E2 stimulation had no significant effect on the rate at which young fibroblasts proliferated or migrated (Table 1); however, in aged fibroblasts, proliferation was increased with E2 treatments vs. untreated control and E2 significantly increased migration rates to those comparable with young cells. Young fibroblasts, seeded onto collagen gels, contracted the gels significantly when exposed to TGF‐β1 (approximately 40% by day 6) (Table 2). Aged fibroblasts also contracted the gels in response to TGF‐β1 by day 6, but to a lesser extent (approximately 14% by day 6). Combinational treatment with E2 and TGF‐β1 restored contraction in aged fibroblasts, comparable to that seen in TGF‐β1‐stimulated young fibroblasts (approximately 35% by day 6). These data suggest E2 treatment was beneficial in the restoration of EGFR‐associated functionality in aged fibroblast cells to rates observable in young fibroblasts, in addition to rescuing reductions in TGF‐β1‐driven contraction. Furthermore, we also used IFN‐γ treatments to demonstrate attenuation of fibroblast functionality, as expected. E2, however, was able to effectively counter‐act the inhibitory effect of IFN‐γ across all three functional assays.

**Table 1:** Proliferation and migration assays in young and aged fibroblasts

Culture conditions		Proliferation (at day 3)		Migration (at day 3)	
		Mean AlamarBlue fluorescence (×10^3^ arbitrary units) ± SEM	Significant? (vs. control) (*P ≤ 0.05, **P ≤ 0.01)	Mean % wound closure (of original wound size) ± SEM	Significant? (vs. control) (*P ≤ 0.05, **P ≤ 0.01)
Young	Control	27.56 ± 3.15	–	99.66 ± 0.13	–
	E2	31.85 ± 2.10	N/S	99.36 ± 0.40	N/S
	IFNγ	19.33 ± 2.13	*	85.92 ± 4.18	*
	E2 + IFNγ	25.35 ± 1.85	N/S	98.40 ± 0.63	N/S
Aged	Control	18.04 ± 1.39	–	56.12 ± 1.09	–
	E2	31.65 ± 1.73	**	87.77 ± 3.28	**
	IFNγ	9.32 ± 0.88	**	32.40 ± 4.85	**
	E2 + IFNγ	24.31 ± 1.90	*	77.26 ± 5.09	**

**Table 2:** Collagen gel contraction assays in young and aged fibroblasts

Culture conditions		Contraction (at day 6)	
		Mean % contraction (of original collagen gel size) ± SEM	Significant? (vs. day 1) (*P ≤ 0.05, **P ≤ 0.01)
Young	Control	23.72 ± 3.09	*
	E2	22.37 ± 2.35	**
	TGFβ1	38.55 ± 2.70	**
	IFNγ	13.47 ± 0.69	N/S
	E2 + TGFβ1	33.35 ± 3.60	*
	E2 + IFNγ	11.10 ± 1.09	N/S
	TGFβ1 + IFNγ	16.08 ± 1.76	*
Aged	Control	6.78 ± 2.30	N/S
	E2	6.08 ± 0.75	*
	TGFβ1	13.79 ± 1.23	*
	IFNγ	7.03 ± 1.06	N/S
	E2 + TGFβ1	33.48 ± 2.77	**
	E2 + IFNγ	8.18 ± 2.26	N/S
	TGFβ1 + IFNγ	6.38 ± 1.89	N/S

## Discussion

Chronic nonhealing wounds are a prominent problem within health care for the elderly and diabetics. While many factors contribute to the persistence of chronic wounds, nonresponsive or senescent‐like fibroblasts at the wound edge play a major role. Our previous research implicated the loss of EGFR as a primary cause for age‐associated resistance to differentiation, which was rescued when miR‐7 was inhibited (Midgley *et al*., 2014). Indeed, dysregulated miR‐7 expression in the skin and sera of patients has been linked to the progression of scleroderma (Etoh *et al*., 2013) and dermatomyositis (Oshikawa *et al*., 2012), providing evidence for the importance of miR‐7 expression and function regarding cutaneous disorders. Here, we report that E2 treatment attenuated miR‐7 expression, positively regulated EGFR mRNA expression, and restored functionality to aging fibroblasts. This was supported by E2‐dependent reductions in miR‐7 transcriptional activity, which involved attenuation of the binding activity, expression, and phosphorylation of the potent miR‐7 activator, STAT1. This research suggests that an E2 treatment regime could be of therapeutic benefit in nonhealing wounds.

An ERE site and several STAT1 and STAT binding sites were identified on the miR‐7 putative TSS. ER binds to ERE with high affinity, in response to E2 (Klinge *et al*., 2001). As the ERE site overlapped with the ISRE and ISGF3G binding sites, it was hypothesized that E2 may potentiate or suppress STAT‐complex binding. The data reported here suggested the latter that a suppression of miR‐7 transcriptional activity was the active mechanism. In contrast, the activation of STAT1 by IFNγ stimulation resulted in potentiation of miR‐7 transcriptional activity; here, we suggest that this was through ISRE, ISGF3G, and upstream STAT1 binding sites. Levels of STAT3 bound to the miR‐7 promoter probe, observed in E2‐treated young fibroblasts, may be causative of increased EGFR expression and activity, as recent research has identified an increase in STAT3 signaling in the presence of EGFR/IL6R association and co‐activation (Wang *et al*., 2013). STAT1 forms complexes with STAT2 and IRF9 to activate ISGF3G/ISRE (Darnell *et al*., 1994); however, STAT3 suppressed STAT1‐dependent gene activation and inhibited STAT1 heterodimer formation (Ho & Ivashkiv, 2006). Furthermore, in tumor cell lines, STAT3 promoted proliferation and cell survival, while STAT1 was antiproliferative and pro‐apoptotic (Pensa *et al*., 2009). The low levels of STAT3 bound to the miR‐7 promoter, in this study, may therefore have an alternative role to transcriptional transactivation of miR‐7; a mechanism for which should be elucidated in future research. Also of interest would be defining the active ER involved, as both ERα and ERβ were identified to associate with the miR‐7 promoter; however, each was reported to have different roles in cell function and wound healing (Gilliver *et al*., 2010).

Chronic inflammation or persistence of bacterial infection is problematic in chronic wounds. In addition, telomerase‐independent senescence and fibroblast dysfunction are regarded as being central to failure of wound closure (Wall *et al*., 2008). The persistence of inflammation and over‐activation of immune‐regulated pathways, such as IFN‐stimulated responses, may contribute to the senescence‐like state of chronic wound fibroblasts, through induction of miR‐7. Increased levels of miR‐7 activity, promoted through stimulation by IFNγ from immune cells such as natural killer T‐cells, macrophages, dendritic cells (Koutoulaki *et al*., 2010; Robinson *et al*., 2010; Nakano *et al*., 2012) or from aging fibroblasts themselves, could be the active mechanism in explaining telomerase‐independent senescence of cells. However, the effects of E2 on IFNγ production may alter between cell types, as shown in adherent and nonadherent splenocytes (Nakaya *et al*., 2006). Taken together, the findings here showed that regulation of IFNγ and IL6 expression by E2 suggests a broader role for E2 in modulating the immune response. Indeed, E2 regulation of macrophage activity has been previously shown (Emmerson *et al*., 2009). Induction or inhibition of immune regulation of fibroblasts could potentially be used as antifibrotic or pro‐healing treatments, respectively.

E2 treatment restored aged fibroblasts to a ‘young state’ in preparation for TGF‐β1‐driven differentiation, through downregulation of miR‐7 and upregulation of EGFR. Fibroblast functionality was almost completely restored, both proliferation and migration benefited from E2. In addition, contraction was also rescued; this could also be explained through miR‐7 targeting the Akt/FAK pathway (Fang *et al*., 2012). However, in aging fibroblasts, the expression of EGFR is lost, suggesting the activity of EGFR‐dependent Akt/FAK would also be diminished. *In silico* evidence suggests that EGFR, Akt, PI3K and c‐Myc are miR‐7 targets; all involved in the EGFR signaling axis (as found in TargetScan and GoDAVID databases). Therefore, miR‐7 could be considered a regulator of fibroblast signaling pathways during wound healing. The JAK/STAT1 pathway provides a novel explanation for the upregulation of miR‐7 observed in aged fibroblast cultures. In conclusion, we propose a mechanism wherein E2 activation of ER results in inhibited STAT1 expression and activity and attenuated binding of STAT1 to the miR‐7 promoter, thereby regulating transcriptional activity and expression of miR‐7. This research implicates E2 as a potential treatment option, in producing beneficial outcomes in the context of fibroblast‐mediated healing, especially in chronic wound conditions.

## Experimental procedures

### Materials and reagents

All reagents were from Sigma‐Aldrich (Poole, UK) unless otherwise stated. Primary antibodies and dilutions for Western blot and immunocytochemistry were polyclonal rabbit anti‐p‐EGFR (1:1000), anti‐p‐ERK1/2, anti‐p‐STAT1, anti‐p‐ STAT3, anti‐STAT1, and anti‐STAT3 (all at 1:2000) from Cell Signalling Technology Inc. (Beverly, MA, USA), polyclonal mouse anti‐GAPDH (1:5000) from Abcam (Cambridge, UK), and anti‐αSMA (1:50) from DAKO (Aachen, Germany). All RT–QPCR reagents were obtained from Life Technologies (Paisley, UK). Other reagents used were E2 (Sigma‐Aldrich) and recombinant human IFNγ and TGF‐β1 (R&D Systems, Abingdon, UK).

### Cell culture

Primary hLF (AG02262; NIA Aging Cell Respiratory, Corriel Institute, Camden, NJ, USA) and hDF (GM23967; NIA Aging Cell Respiratory) were cultured in 10% fetal calf serum (FCS) and Dulbecco's modified Eagle's medium (DMEM)/F‐12 Ham's medium as previously described (Midgley *et al*., 2014). All cells were growth arrested in serum‐free (SF) medium for 48 h before use in experiments, unless otherwise stated. Young fibroblasts were cells at passages 6–8 [population doubling level (PDL) 15–20], while aged fibroblasts were cells at presenescent late passages 14–15 (PDL 26–28); cells underwent senescence at PDL 30 as determined by growth curves (data not shown). All experiments were performed on confluent cell monolayers except for those experiments using antibody visualization (optimal confluence was approximately 70%). Cells were differentiated to myofibroblasts by incubating fibroblast cultures in SF medium containing 10 ng mL^−1^ TGF‐β1 for 72 h.

### Real time and reverse transcription–quantitative polymerase chain reaction

Isolation of RNA and cDNA reverse transcription was as previously described (Midgley *et al*., 2014). Reverse transcription–quantitative polymerase chain reaction (RT–qPCR) was performed according to the TaqMan or Power SYBR Green Master Mix kit protocols and using the ViiA7 Fast Real‐Time PCR System (Life Technologies). Ribosomal RNA (rRNA) was used as an endogenous control. MicroRNA‐RT and qPCR were performed for miR‐7 according to TaqMan MicroRNA Assay Kits (Life Technologies). EGFR, αSMA, HAS2 and STAT1 primers were commercially available. Primers (5′–3′) for IFNγ (forward: ACAGGGAAGCGAAAAAGGAGT; reverse: TATTGCAGGCAGGACAACCAT), IL6 (forward: TGAACTCCTTCTCCACAAGCG; reverse: TGGAATCTTCTCCTGGGGGTA), STAT3 (forward: ATCCTGGTGTCTCCACTGGT; reverse: GCTACCTGGGTCAGCTTCAG) and EDA‐FN (forward: ACAGTCAGTGTGTGGTTGCCTT; reverse: TTCAGGTCAGTTGGTGCAGG) were custom designed. All custom primer pairs were tested for efficiency by log_10_ standard dilutions, where ≥90% was deemed sufficient.

### Western blot analysis

Total protein was extracted in RIPA lysis buffer containing 1% protease inhibitor cocktail (PIC), 1% PMSF, and 1% sodium orthovanadate (Santa Cruz Biotechnology, Santa Cruz, CA, USA). Protein was quantified before SDS‐PAGE and transfer to nitrocellulose. Membranes were blocked with 5% BSA/0.5% Tween‐20/PBS for 1 h, RT, followed by incubation with primary antibodies diluted in 1% BSA/0.1% Tween‐20/PBS, overnight at 4 °C. Following wash steps, membranes were incubated in secondary anti‐rabbit/mouse IgG HRP conjugate (Cell Signalling Technology; 1:5000 dilution, 1% BSA/0.1% Tween‐20/PBS). Detection was performed using ECL reagent (GE Healthcare, Buckinghamshire, UK) and exposure to X‐ray film (GE Healthcare).

### Chromatin immunoprecipitation

Protein‐DNA was cross‐linked in 0.75% v/v formaldehyde (10 min, RT) followed by 125 mm w/v glycine (5 min, RT). Cells were harvested into 1 mL ice‐cold PBS and pelleted at 1000 ***g*** for 5 min before resuspension in FA lysis buffer (50 mm HEPES pH7.5, 140 mm NaCl, 1 mm EDTA pH 8, 1% Triton‐X100, 0.1% sodium deoxycholate, 0.1% SDS, 1% PIC and 1% PMSF). Lysates were sonicated to shear DNA to approximate fragments of 1000 bp and centrifuged for 30 s, 8000 ***g*** at 4 °C. Supernatant was transferred to new Eppendorfs, and 50 μL was removed for use as an input sample. A known volume of 25 μg of protein in RIPA buffer was immunoprecipitated (IP) using anti‐ERα or anti‐ERβ antibody‐linked protein A/G beads (pre‐absorbed with sonicated single‐stranded herring sperm DNA). IP was completed with an overnight rotating incubation at 4 °C. The bead complexes were centrifuged for 1 min at 2000 ***g***, and the supernatant was removed. Beads were washed with wash buffer (0.1% SDS, 1% Triton‐X100, 2 mm EDTA pH8, 150 mm NaCl, 20 mm Trizma base pH8) three times and once with final wash buffer (wash buffer containing 500 mm NaCl). Protein–DNA was eluted with elution buffer (1% SDS, 100 mm NaHCO_3_) at RT for 1 h with continuous rotation. Supernatant was protein digested with 50 μg/mL proteinase K at 65 °C for 5 h. DNA was extracted with phenol–chloroform and precipitated in ethanol–glycogen, before resuspension in nuclease‐free H_2_O. DNA amplified from the ChIP elute was visualized on EtdBr–agarose gel.

### Luciferase reporter plasmid generation

A 424‐bp miR‐7 promoter insert was PCR amplified using Phusion DNA polymerase (New England Biolabs, Herts, UK). Primers (5′–3′): forward CTTGACAGGTTTAGGGAGCGT and reverse AGGTCCCCAAAAGGTTGAGAC (with KpnI and XhoI endonuclease restriction sites, respectively). Inserts were ligated into KpnI‐ and XhoI‐digested pGL3Basic (pGL3b) Vector using T4 DNA ligase (New England Biolabs) overnight at 16 °C. The pGL3b Vector containing the promoter insert (pGL3b‐miR‐7) was heat‐shock‐transformed into one‐shot competent *Escherichia coli* (New England Biolabs) and grown on ampicillin–agar. Plasmid DNA was purified from colonies according to the Miniprep Kit protocol (Sigma‐Aldrich). Cloned pGL3b‐miR‐7 was confirmed with DNA sequencing (DNA Sequencing Core, Cardiff University, Cardiff, UK).

### Transfection and luciferase reporter analysis

Plasmid pGL3b‐miR‐7 transfections were completed using Lipofectamine LTX (Life Technologies) following optimization. Reporter analysis was performed 72 h post‐transfection using the Dual‐Luciferase reporter assay kit (Promega, Southampton, UK) and detected with a FLUOstar OPTIMA plate reader (BMG Labtech, Ortenberg, Germany). Renilla luciferase was co‐transfected with pGL3b‐miR‐7 and used as a control for transfection efficiency and normalization.

### Electromobility shift/supershift assay

A 424‐bp miR‐7 promoter fragment was PCR amplified from genomic DNA using Phusion DNA polymerase (New England Biolabs), biotinylated forward primer; 5′‐CTTGACAGGTTTAGGGAGCGT‐3′ and reverse primer; 5′‐AGGTCCCCAAAAGGTTGAGAC‐3′. Nuclear extraction was performed according to the following protocol: Total cell lysate was resuspended in hypotonic buffer (20 mm Tris‐HCl pH7.4, 10 mm NaCl, 3 mm MgCl2) on ice for 15 min. About 10% NP40 detergent was added and samples vortexed. Homogenate was centrifuged for 10 min at 1000 g at 4 °C, and the supernatant (cytoplasmic fraction) was removed and frozen for future use. The pellet was resuspended in cell extraction buffer (100 mm Tris‐HCl pH7.4, 2 mm Na_3_VO_4_, 100 mm NaCl, 1% Triton X‐100, 1 mm EDTA, 0.1% SDS, 1 mm NaF, 0.5% deoxycholate, 20 mm Na_4_P_2_O_7_, 1% PIC) and incubated on ice for 30 min with regular mixing. Samples were centrifuged at 14 000 ***g*** at 4 °C for 30 min, and the supernatant (nuclear extract) was transferred to fresh Eppendorfs. Protein was quantified and EMSA performed according to the following protocol: a 10 μL binding reaction was set up, containing 100 ng protein, 50 fmol μL^−1^ biotinylated‐DNA probe, 1 μg μL^−1^ poly dI:dC, 50 μg μL^−1^ BSA, 0.1 m DTT, 0.1 m MgCl2, buffer D (20 mm HEPES‐KOH pH7.9, 20% glycerol, 0.2 mm EDTA, 0.1 m KCl, 0.5 mm PMSF, 1 mm DTT), and H_2_O. The reaction was incubated at 30 °C for 1 h before the samples were run on a 4.5% nondenaturing acrylamide gel at 150 V in ice‐cold 0.5× TBE buffer. The DNA was transferred in 0.5× TBE at 30 mA for 1 h, onto nylon membranes, UV‐cross‐linked and blocked with 5% milk. Streptavidin–HRP was incubated with the membrane for 30 min (darkness, RT). DNA bands were detected using ECL reagent (GE Healthcare, Amersham, UK) and exposed to X‐ray film (GE Healthcare). For supershift assays, 1 μg of anti‐STAT1 or anti‐STAT3 antibody was added to the binding reaction.

### Immunocytochemistry

Cells were fixed in 4% paraformaldehyde for 10 min at room temperature. To ensure visualization of intracellular proteins, fixed cells were permeabilized with 0.1% Triton X‐100 in PBS (10 min, RT). Slides were washed with PBS and then blocked in 1% BSA/PBS for 30 min prior to a wash step with 0.1% BSA/PBS. Subsequently, the slides were incubated with mouse anti‐αSMA antibody diluted in 0.1% BSA/PBS overnight at 4 °C. Following a further wash step, slides were incubated with secondary anti‐mouse IgG/AlexaFluor 488 (Invitrogen, Paisley, UK) in 0.1% BSA/PBS (1 h, RT in darkness). Cells were then mounted and analyzed by fluorescence microscopy. Alternatively, slides were incubated with phalloidin‐FITC (Sigma‐Aldrich; 2 h, RT in darkness), to visualize F‐actin.

### Proliferation assay

Cells were seeded at approximately 5 × 10^4^ per mL for 24 h before growth arrest. AlamarBlue (Life Technologies) was added to media at 10% v/v at each 24‐h time‐point, and incubated for 1 h; aliquots of conditioned AlamarBlue/medium were transferred to clear 96‐well plates and fluorescence was measured at excitation wavelength of 540 nm and emission at 590 nm on a FLUOstar OPTIMA plate reader (BMG Labtech). Measurements were made over a course of 72 h of cellular treatments and are expressed as arbitrary fluorescence units.

### Migration/scratch‐wound healing assay

Scratching quiescent cell monolayers with sterile 200 μL pipette tips generated linear denuded areas. The cells were gently washed with PBS to remove detached cells and then replenished with fresh SF medium containing appropriate cytokine treatments. The wound size was photographed every 24 h, up to 72 h or until closure, using an Axiovert 100 m inverted microscope fitted with a digital camera (ORCA‐1394; Hamamatsu Photonics, K.K., Hamamatsu, Japan). Measurements were obtained using imagej (NIH Software, Bethesda, MD, USA). Data are expressed as % reduction in wound area, compared to wound area at 0 h.

### Collagen gel contraction assay

Type I collagen was extracted from rat‐tail tendon as previously described (Cawston & Barrett, 1979). Approximately 2.5 × 10^5^ per mL fibroblasts were seeded onto preformed collagen lattices (2.5 mL 20% FCS‐DMEM, 500 μL 0.1 m NaOH and 1 mg mL^−1^ type I collagen, total volume of 5 mL). Fibroblast populated collagen lattices (FPCLs) were maintained at 37 °C, in a 5% CO_2_ atmosphere for 1 h, for collagen polymerization to occur. FPCLs were gently detached from the plate edges and resuspended in SF medium containing appropriate cytokine treatments. FPCLs were measured at days 1, 3 and 6 after initial lattice fabrication. The average FPCL contraction values were obtained from imagej (NIH Software) analysis and are expressed as % reduction in gel diameter, compared to the gel diameters, at 0 days.

### *In silico* analysis

Putative TSS and potential transcription factor‐binding regions were analyzed using miRStart (National Chiao‐Tung University, Hsinchu, Taiwan) and Genomatic MatInspector (Genomatix Software GmbH, Munich, Germany).

### Statistical analysis

Densitometrical analysis was by imagej (NIH Software). Graphical data are displayed as means ± SEM. One‐ or two‐way ANOVAs and post‐test Bartlett's were used to determine statistical differences across multiple data groups. The unpaired two‐tailed Student's *t‐test* was used to identify statistical significance. Data were analyzed using the software GraphPad Prism version 4.0a (GraphPad Software Inc., La Jolla, CA, USA) and *P ≤ 0.05 and **P ≤ 0.01 were considered significant.

## Funding

This work was supported and funded in part by the Kidney Wales Foundation and a studentship from Research into Aging/Age UK (Grant 358) (to A.C.M. and R.S.).

## Conflict of interest

None declared.

## Author contributions

A.C.M. wrote the manuscript, designed/performed experiments, and collected/analyzed the data. G.M. assisted with experiments and provided resources/reagents. R.S. and A.O.P. contributed equally to the manuscript, supervised the project, and edited the manuscript. All authors discussed results and implications of data throughout all stages of the project.

## Supporting information


**Fig. S1** (A–B) Fibroblast cultures were assessed for proliferative capacity under the indicated treatments over the course of 3 days, using the AlamarBlue assay as described under methodology. (C–D) Migration ability was determined by scratch‐wound assay, and under indicated treatments.

**Fig. S2** Fibroblast contraction was examined through collagen gel contraction assays.
