# Supplementary figures and images for: 17β‐estradiol ameliorates age‐associated loss of fibroblast function by attenuating IFN‐γ/STAT1‐dependent miR‐7 upregulation
Source: Aging Cell. 2016 Mar 2;15(3):531–41. doi: 10.1111/acel.12462 (PMC4854905; doi:10.1111/acel.12462)

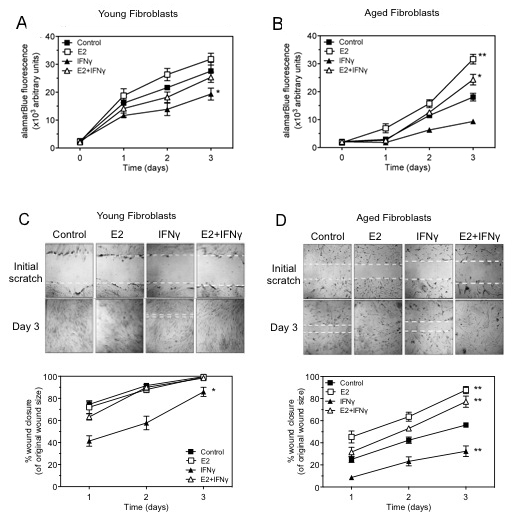

Supplement: Supplementary file 1 — Fig. S1 (A–B) Fibroblast cultures were assessed for proliferative capacity under the indicated treatments over the course of 3 days, using the AlamarBlue assay as described under methodology. (C–D) Migration ability was determined by scratch‐wound assay, and under indicated treatments. [file ACEL-15-531-s001.jpg]

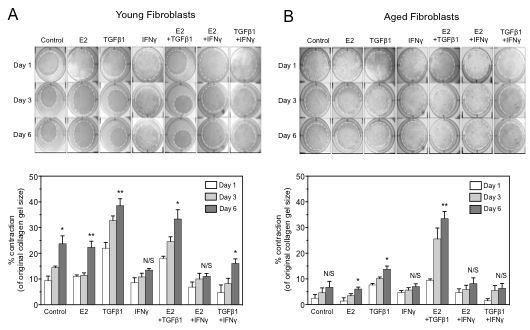

Supplement: Supplementary file 2 — Fig. S2 Fibroblast contraction was examined through collagen gel contraction assays. [file ACEL-15-531-s002.jpg]
